# Metastable Amorphous Dispersions of Hydrophobic Naphthalene Compounds Can Be Formed in Water without Stabilizing Agents via the “Ouzo Effect”

**DOI:** 10.1021/acs.jpcb.3c03885

**Published:** 2023-09-12

**Authors:** Julie M. Belanger, Joseph A. Cirilo

**Affiliations:** King’s College, Department of Chemistry and Physics, 133 N. River St., Wilkes-Barre, Pennsylvania 18711, United States

## Abstract

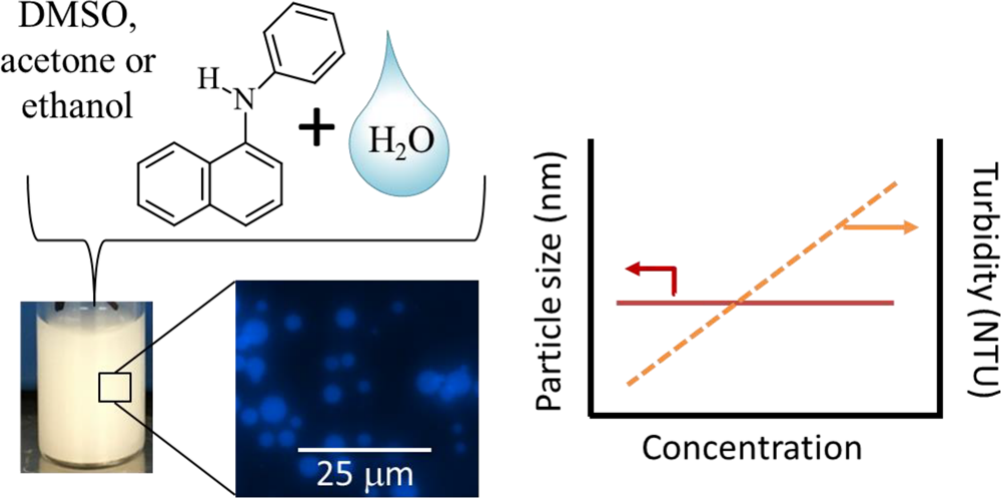

Hydrophobic molecules dissolved in water-miscible organic solvents are used in vitro for biological membrane studies and for testing of potential pharmaceuticals in high-throughput screenings. When these solutions are introduced into an aqueous environment, it is possible that metastable “ouzo-like” dispersions form from liquid–liquid phase separation. It is therefore hypothesized that when solutions of naphthalene compounds in water-miscible solvents are added to water, metastable dispersions will form. Millimolar solutions of naphthalene, *N*-phenyl-1-naphthylamine (NPN), 1-aminonaphthalene, 1-iodonaphthalene (INAP), 1,4-dimethoxynaphthalene, and 1-naphthol were prepared in either dimethyl sulfoxide, ethanol, or acetone at concentrations similar to those used in biological membrane studies. Each solution was diluted 10-fold in water. Particle formation was characterized by qualitative observations, dynamic light-scattering, nephelometry, and optical microscopy. It was discovered that two of the compounds tested made metastable dispersions: INAP and NPN. The initial particle sizes were ∼400 nm (radius), with turbidity ranging from 1,000 to 20,000 NTU, depending on the initial concentrations used. Fluorescence microscopy imaging showed spherical particles that do not aggregate while under observation. Slow-nucleating crystallization occurs over days, presumably from a heterogeneous nucleation process. The formation of these dispersions has implications for in vitro delivery of hydrophobic molecules to biological membranes.

## Introduction

Typically, precipitation or bulk phase separation occurs when hydrophobic compounds are dissolved in a water-miscible solvent and then are added to water. This is a fundamental result of intermolecular forces and solvation; the water–solvent interaction is more favorable, and the hydrophobic compound is excluded from solution, resulting in bulk phase separation (i.e., precipitation of a solid). Synthetic polymer purification, for example, takes advantage of this process to isolate polymer solids (such as polystyrene) from an organic solvent (toluene) when it is added to methanol (a good solvent for toluene but not polystyrene). Based on thermodynamics, the process of aqueous solvation of the water-miscible solvent will result in the formation of a precipitate as a spontaneous event. While fundamentally this always holds true, there are systems in which the formation of crystalline precipitate is delayed due to a stabilizing kinetic barrier, resulting in a metastable dispersion.^1^

Dispersions resulting from what has been coined the “ouzo effect” are created when a water-miscible solvent (such as ethanol, acetone, DMSO, or NMP) is used to dissolve a hydrophobic molecule, and this solution is then added to water to create a milky-white suspension. This “ouzo effect”, derived from the Greek drink of the same name,^2^ results in a stable dispersion without the need for added stabilization agents. Such dispersions have been referred to as colloidal dispersions,^3^ liquid–liquid droplet dispersions,^2^ liquid–liquid phase separation (LLPS),^4^ microemulsions,^5^ nanoparticle suspensions,^6,7^ amorphous solid dispersions,^8,9^ or nanometric droplets.^10^ These dispersions are being explored for their use in the drug delivery of active pharmaceutical ingredients.^6−8^ The particles that are suspended in these dispersions result from the initial formation of solution droplets (containing solvent and hydrophobic compounds) that fairly rapidly create supersaturated droplets of solution via solvent-shifting.^3^ These droplets can then grow in size over time by Ostwald ripening until a stable dispersion is created. The size and distribution of these particles can be tailored depending on the excess “oil-to-solvent ratio” and temperature.^2^ The stability of these dispersions can be tailored by the addition of stabilizers, dilution with water, or evaporation of the organic water-miscible solvent once the dispersion is formed.^3^ Eventual bulk phase separation occurs, resulting in the crystallization of solid solutes or “creaming” of liquid solutes. Even so, with proper tailoring of the solvents and solute(s) used, the emulsions can be made stable for days.

Polar water-miscible solvents, such as ethanol, acetone, and DMSO, are routinely used to dissolve compounds that are nonpolar or have limited water solubility. These solutions can then be added to an aqueous environment to disperse the compound in an attempt to avoid immediate precipitation and crystallization. Such solutions have been used in vitro to introduce water-insoluble naphthalene-derived compounds into micelles,^11^ lipid membranes found in cells,^12,13^ and enveloped viruses.^14−18^ In vivo studies have also been conducted using acetone and DMSO to deliver hydrophobic compounds to zebra fish.^19^ For example, in a typical in vitro protocol, cells or purified virus preparation is suspended in aqueous buffer, and the hydrophobic compound (in mM concentration in DMSO) is introduced with mixing. The use of water-miscible solvents as a vehicle to deliver hydrophobic compounds through the aqueous environment suggests that the formation of “ouzo-like” dispersions in these scenarios is possible but likely to have gone unnoticed due to the already existing turbidity in cell cultures and virus isolates in vitro. The formation of dispersions has been noted in drug development screening of poorly water-soluble compounds which can lead to erroneous results in, for example, protein inhibition assays.^4,20^ In these assays, the candidate compounds are first dissolved in an organic solvent and then added to an aqueous buffer containing the drug target. Depending on the solubility of the compound and the concentration used, a dispersion may form.

The metastable nature of “ouzo-like” dispersions, when contrasted with simple precipitation of the hydrophobic compounds in an aqueous environment, has potential implications on biological studies that use these solutions to deliver compounds to biological membranes. Previous research using hydrophobic drugs has shown an enhancement in drug membrane transport for systems that undergo liquid–liquid phase separation.^4,20^ Should these “ouzo-like” dispersions form, it is possible that a similar enhancement in the delivery of these compounds to the membrane may occur, highlighting the importance of understanding this basic phenomenon in these new systems. Herein, we describe the discovery and characterization of the formation of a metastable dispersion when certain naphthalene-based compounds are dissolved in either a polar aprotic solvent (DMSO or acetone) or a polar protic solvent (ethanol) and are introduced into distilled water at concentrations similar to those used in in vitro biological studies. The naphthalene compounds chosen for our study are based on their similarity to those already explored in previous studies for viral inactivation that have been shown to permeate into biological membranes.^14,16,21^ Additional naphthalene compounds with varying polarities were also chosen to test the formation and stability of the dispersions that were formed.

## Materials and Methods

### Creation of Dispersions

*N*-Phenyl-1-naphthylamine (NPN, Acros, 98%, CAS 90-30-2) supplied as a dark brown solid was sublimed at 55 °C and 0.5 Torr to yield an off-white powder. NPN was stored away from light in a desiccator when not in use. Naphthalene (Aldrich, 99%), 1-aminonaphthalene (Sigma, 98%), 1-iodonaphthalene (INAP, liquid, Acros, 97.5%), 1,4-dimethoxynaphthalene (DMN, Aldrich), and 1-naphthol (Acros, 99%) were used as supplied. Dimethyl sulfoxide (DMSO) (Sigma-Aldrich, Sure-seal, anhydrous, 99.9%) was aliquoted into glass vials at room temperature and stored for up to 2 weeks. Acetone (Sigma-Aldrich, 99.5%) and ethanol (Pharmco-Aaper, absolute, anhydrous, ACS/USP grade) were used as supplied. Solutions of each naphthalene compound in DMSO, acetone, or ethanol (8, 16, 32, and 64 mM) were made immediately before use. Dispersions were prepared by the quick addition of the naphthalene compound in DMSO (or acetone, or ethanol) solution into distilled water as a 10-fold dilution (for example: 100 μL of NPN/DMSO solution added to 900 μL of water) in glass vials, followed by immediate vortex mixing for 3 s. For samples that made dispersions, the result was cloudy white in appearance with no crystalline precipitate. Note that the dispersions throughout are referred to by the starting naphthalene compound/DMSO or naphthalene compound/acetone solution concentration. For example, an “8 mM NPN/DMSO” sample implies the starting solution used to generate the dispersions was 8 mM NPN dissolved in DMSO before the addition to water, resulting in a 10-fold dilution.

### Fluorescence Microscopy

Samples were placed as a drop (10 to 50 μL) onto a glass slide and imaged using the bright field and fluorescence settings of an EvoFL imaging system (Life Technologies, Thermo Fisher Scientific). Fluorescent images were taken using the “DAPI” setting (λ_max_ excitation = 367 nm, excitation range = 350 – 410 nm, experimentally determined) on the microscope. NPN fluorescence (λ_ex_ = 345 nm, λ_em_ = 436 nm) of the droplets was observed as blue spheres on a black background. The dispersions were stored in screw-top vials, covered in foil to protect from light, at 25 °C. For the initial measurements (*t* = 0) the sample was used within an hour of being prepared. For the 24 and 48 h samples, fresh sample was removed from the stored vials and placed on the glass microscope slide for imaging.

### Determination of Concentration for Onset of Colloid Formation

Measurements of scattering due to turbidity were taken by using a Horiba Scientific Dual-FL spectrofluorometer as a nephelometer. An “excitation” wavelength of 650 nm was used, and the intensity of 650 nm light scattered at 90° (at the “emission” detector) was measured. Fresh 32 mM solutions of NPN, INAP, or DMN were prepared in DMSO, acetone, or ethanol. Amounts varying from 0 to 200 μL of each of these solutions were added to 20.0 mL of deionized water in a fresh glass scintillation vial. Note that “actual” volumes used were corrected volumes according to calibration curves for the pipettor used with each solvent. The vial was vortex mixed to make a uniform dispersion, and measurements were taken immediately after mixing. A volume of 0.50 mL of each sample was placed into a quartz microcuvette (Starna cells, catalog #18F-Q-10). Cuvettes were used with their long axes oriented perpendicular to the incident beam. All measurements were taken using 16 scans, 0.58 nm resolution, and a 0.01 s integration time. Raw intensities were recorded as counts on the instrument. Each data point was performed in true triplicate (fresh solution made and a fresh scintillation vial used each time). Data were plotted as micrograms of solute (NPN, INAP or DMN) per milliliter of total solution. Error bars reflect plus or minus one standard deviation for true triplicate samples (new solutions made for each replicate), and the *x*-intercept was determined by using the LINEST function in Excel to determine the standard uncertainty in *x*.

### Turbidity Measurements

Turbidity measurements were taken by using a Horiba Scientific Dual-FL spectrofluorometer as a nephelometer. An “excitation” wavelength of 650 nm was used, and the intensity of 650 nm light scattered at 90° (at the “emission” detector) was measured. Note that at this wavelength the turbidity standards and the experimental samples did not absorb or fluoresce. Linear calibration curves were created using the scattered light intensities of a freshly suspended 1000 NTU turbidity standard (Ricca Chemical, 1000 NTU standard, Fisher Scientific catalog #8825-16) and ten sequential 2-fold dilutions of the standard. All measurements were taken using 16 scans, 0.58 nm resolution, and 0.01 s integration time. Quartz microcuvettes (Starna cells, catalog #18F-Q-10) were used with their long axis oriented perpendicular to the incident beam. Dispersions made with 8, 16, 32, or 64 mM of NPN in DMSO (or other solvent specified) suspended in water were each subsequently diluted with water to an effective 0.8 mM total NPN concentration in the dispersion before measuring turbidity (for example, 100 μL of the aqueous dispersion produced using 16 mM NPN/DMSO was added to 100 μL of water). This created solutions with turbidities within the linear calibration curve created by using the turbidity standard. Note that raw scattering intensities were also collected for the concentrated samples (for example: the starting dispersion made with 64 mM NPN/DMSO and dilutions of this dispersion to 0.8 mM total NPN) and were compared to ensure that the intensities were linear upon dilution. NTU values then for concentrated (original) samples could be calculated by using the calibration curve and multiplying by the dilution factor.

### Stability Determination

Stability over 2 days was determined upon visual inspection of triplicate experiments using 0.5 mL of dispersion per vial. Vials were visually inspected for stable dispersion and the presence of a crystalline precipitate (or liquid droplets for 1-iodonaphthalene, a liquid) at various time points. Vials were resuspended by vortex mixing for 3 s prior to observations, as the dispersion would settle over time. The vials assessed were also used for turbidity and particle sizing data and were, therefore, periodically opened and closed over this time period. Vials with stable dispersion and no visible crystals were given a value of “1”; vials with dispersion and some visible precipitated crystals were given a value of “0.5”, and vials containing no dispersion and only crystals (complete precipitation) were given a value of “0”. The percentage of stable dispersions per time point was determined as the sum of these values divided by the number of vials (*n*) used. The experiment was done in true triplicate, with two to four vials for each experiment, where the total number of vials used varied from 10 vials (*n* = 10) initially, as some samples were used up for turbidity and particle sizing experiments, with the smallest remaining number of vials being six (*n* = 6).

### Dynamic Light Scattering

Particle sizing of the dispersions was done using a Wyatt Dynapro Nanostar light-scattering detector using dynamic light-scattering (DLS) measurements to determine the hydrodynamic radii. Initially, all NPN in DMSO dispersions was made at their specified starting concentrations and then diluted to 8 mM with water, to provide samples of similar turbidity before particle sizing. (For example, the 16 mM sample was diluted 2-fold with the addition of water before DLS measurements were taken.) Comparison to undiluted samples of NPN/DMSO showed particle size results similar to those of the diluted dispersions. Therefore, all other measurements for the additional compounds and hydrotropes tested were done at their original concentrations, as dilution was determined to have no effect on particle size for a given dispersion. For each measurement, 10 μL (microliters) of sample was placed into a plastic microcuvette (Wyatt Technology, part #WNDMC). Measurements were taken at 25.00 °C, with 30 measurements taken in total (grouped in 3 groups of 10 measurements) per sample concentration. DYNAMICs software was used with the method of Regularization to fit particle sizes and distributions. Particle sizes are reported herein as the averaged particle radius in nanometers of true experimental triplicates (solutions prepared fresh on different days, for a total of 9 groups of 10 measurements) for each sample concentration measured.

## Results and Discussion

### Dispersion Stability and Appearance

Various naphthalene compounds were tested for their ability to form metastable dispersions after the compounds were dissolved in DMSO, ethanol, or acetone and then added to water (Table 1). Two compounds tested, 1-aminonaphthalene and 1-naphthol, in DMSO, ethanol, or acetone formed clear solutions upon addition to water. Naphthalene solutions in these solvents, however, formed an immediate crystalline precipitate upon addition to water. Conversely, it was found that DMSO, ethanol, or acetone solutions of 1-iodonaphthalene (INAP), 1,4-dimethoxynapthalene, and *N*-phenyl-1-naphthylamine (NPN) all formed dispersions when added to water for the concentrations tested. When toluene or chloroform was used with these compounds at similar concentrations, bulk phase separation occurred without the formation of dispersions (data not shown).

**Table 1:** Testing of the Formation of Dispersions^a^

Compound Tested	Result When Added to Water
1-aminonaphthalene	clear solution
1,4-dimethoxynaphthalene	cloudy white dispersion
1-iodonaphthalene (INAP)	cloudy white dispersion
naphthalene	crystalline precipitate
1-naphthol	clear solution
*N*-phenyl-1-naphthylamine (NPN)	cloudy white dispersion

a Each compound was dissolved in either DMSO, ethanol, or acetone (as 8 mM, 16 mM, 32 mM, or 64 mM solutions) and then diluted 10-fold in water.

The stability of these dispersions that were formed varied depending on the compound and initial polar aprotic or polar protic solvent used (Table 2). The 1,4-dimethoxynaphthalene in acetone very rapidly precipitated as crystals after the initial formation of a uniformly turbid dispersion. When this compound was tested using DMSO starting solutions, it crystallized more slowly over the course of 30 min. The INAP and NPN dispersions were more stable over days, as shown. Generally, when the starting solutions were made from acetone, the dispersions were less stable over time and formed precipitates faster than those for the DMSO solutions of comparable concentration. It should also be noted that the dispersions were more stable over time in glass vials with screw top lids versus plastic microfuge tubes. This suggests that the stability of these dispersions over time partially depends on the interactions (physical or chemical) with the sample vessel. In fact, periodically a prepared NPN/DMSO sample, when left in an unopened glass vial, would last weeks, allowing for periodic resuspension without crystallization occurring. This was usually an exception but does further support heterogeneous nucleation as the cause of the crystalline precipitate.

**Table 2:** Stability of Naphthyl-Compound Dispersions over Time

		Percentage of Vials with Visible Dispersion				
Solvent	Starting Stock Concentration	*t* = 0	*t* =5 min	*t* =30 min	*t* =24 h	*t* =48 h
**1-aminonaphthalene**^a^						
**DMSO**	8 mM - 64 mM	0%	—	—	—	—
**acetone**	8 mM - 64 mM	0%	—	—	—	—
**ethanol**	8 mM - 64 mM	0%	—	—	—	—
**1,4-dimethoxynaphthalene**						
**DMSO**	64 mM	100%	100%	50%	0%	—
	32 mM	100%	100%	50%	0%	—
	16 mM	100%	100%	25%	0%	—
	8 mM	100%	100%	25%	0%	—
**acetone**	8 mM - 64 mM	100%	0%	—	—	—
**ethanol**	8 mM - 64 mM	100%	0%	—	—	—
**1-iodonaphthalene**						
**DMSO**	8 mM - 64 mM	100%	100%	100%	100%	100%
**acetone**	64 mM	100%	100%	100%	80%	25%
	32 mM	100%	100%	100%	90%	80%
	16 mM	100%	100%	100%	100%	80%
	8 mM	100%	100%	100%	100%	80%
**ethanol**	8 mM - 64 mM	100%	100%	100%	100%	100%
**naphthalene**^b^						
**DMSO**	8 mM - 64 mM	0%	—	—	—	—
**acetone**	8 mM - 64 mM	0%	—	—	—	—
**ethanol**	8 mM - 64 mM	0%	—	—	—	—
**1-naphthol**^a^						
**DMSO**	8 mM - 64 mM	0%	—	—	—	—
**acetone**	8 mM - 64 mM	0%	—	—	—	—
**ethanol**	8 mM - 64 mM	0%	—	—	—	—
*N***-phenyl-1-naphthylamine (NPN)**						
**DMSO**	64 mM	100%	100%	100%	30%	22%
	32 mM	100%	100%	100%	29%	9%
	16 mM	100%	100%	100%	59%	25%
	8 mM	100%	100%	100%	68%	44%
**acetone**	64 mM	100%	100%	100%	38%	25%
	32 mM	100%	100%	100%	65%	50%
	16 mM	100%	100%	100%	95%	75%
	8 mM	100%	100%	100%	100%	85%
**ethanol**	64 mM	100%	100%	100%	58%	17%
	32 mM	100%	100%	100%	100%	58%
	16 mM	100%	100%	100%	100%	75%
	8 mM	100%	100%	100%	100%	75%

a Formed clear solutions at the concentrations used.

b Formed crystalline precipitate immediately upon addition to water.

**Figure 1:**
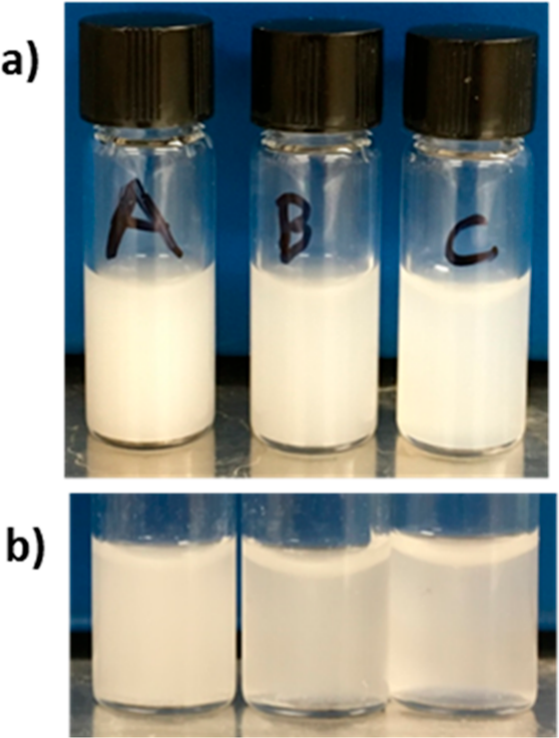
Glass vials containing dispersions of varying concentrations show varying turbidity. (a) Samples immediately after formation (*t* = 0) and (b) samples 24 h later after resuspension by vortex mixing. A = 32 mM, B = 16 mM. and C = 8 mM NPN in DMSO samples.

The dispersions that were formed were very turbid, milky white dispersions (see Figure 1). These aqueous dispersions are referred to herein as “naphthalene compound/solvent” (e.g., “NPN/acetone”). The differences in the cloudiness (or turbidity) of the samples can be seen. The vials that contained a greater starting concentration of NPN appeared to be more turbid than the lower concentration samples. In these samples, the same volume of NPN solution was added, indicating that the turbidity is a result of the NPN concentration and not the total volume of solution added. Over time, the turbidity of the samples decreased.

**Figure 2:**
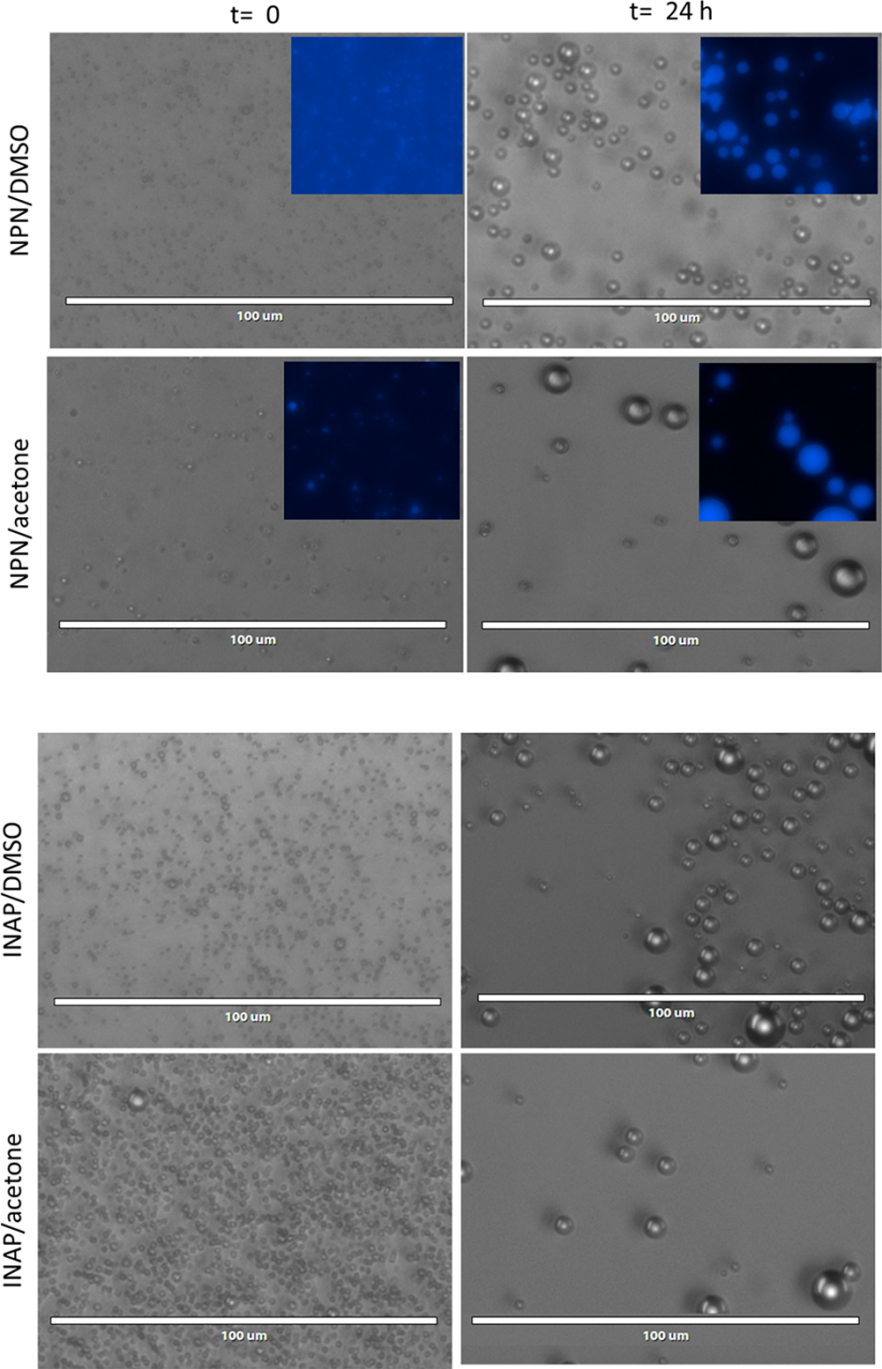
Optical microscopy shows spherical droplets. Imaging was done on the dispersions at *t* = 0 and *t* = 24 h. Insets show fluorescent images taken of the respective NPN samples. Note that INAP is not fluorescent. All starting solute concentrations are 64 mM. Scale bars are 100 μm, with the fluorescent insets also to scale.

Initially it was assumed that these cloudy dispersions were due to the precipitation of the NPN solid (or INAP liquid) compounds forming small crystals (or droplets of pure liquid for INAP) since no additional stabilizing agents were added to the solution. While it seemed highly unlikely that colloids would form due to the solubility of DMSO (or acetone) in water and the immiscibility of NPN or INAP in water, microscopic analysis shows that the formation of crystalline NPN precipitate, or droplets of pure INAP, does not initially occur (see Figure 2). The white dispersions that form contain spheres that move about freely in the solution due to random motion. These spheres bounce off one another and do not aggregate, nor do they readily coalesce during the time frame of observation in the microscope. Settling does eventually occur over several hours; however, the dispersions can be resuspended by vortex mixing. The size of the droplets within these suspensions does change over time, as noted by the two time points, 0 and 24 h, shown in the microscopy images (Figure 2). The fluorescent properties of NPN indicate that the spheres contain NPN. Similar results were seen for all NPN/solvent and INAP/solvent samples suspended in water (fluorescence data not shown).

**Figure 3:**
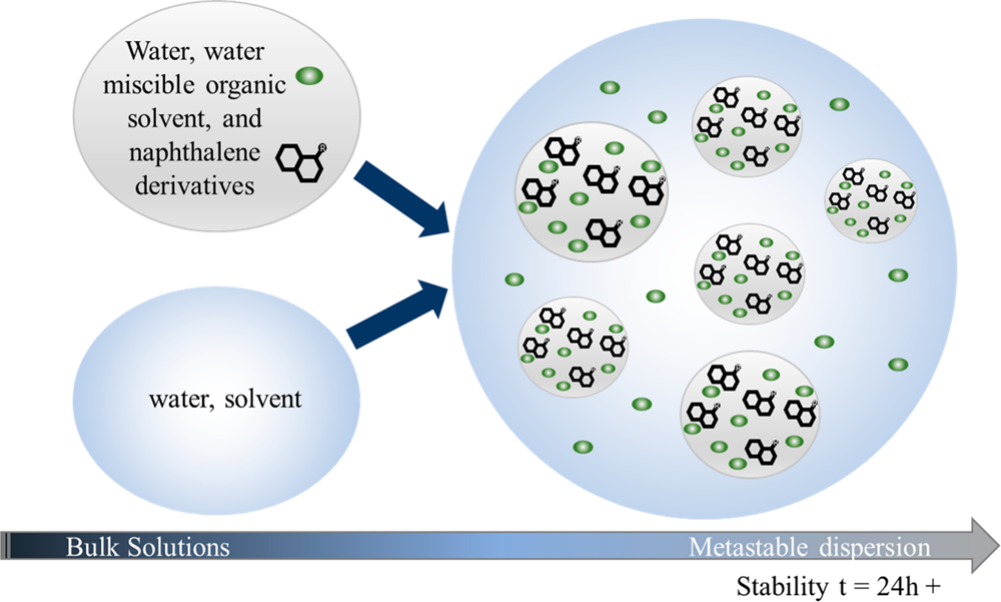
According to the “ouzo effect”, metastable supersaturated droplets are formed in the aqueous milieu within water-rich and water-poor domains.

This is consistent with theories supporting the formation of colloidal dispersions of amorphous droplets by the “ouzo effect”, where it is assumed that the particles seen are actually the result of the initial formation of a droplet containing a supersaturated solution.^3^ This explains the apparent “liquid” nature of these droplets when observed; they are most likely supersaturated droplets of NPN (or INAP) in either DMSO or acetone that are suspended in water (Figure 3).

### Onset of Colloid Formation and Turbidity Determination

**Figure 4:**
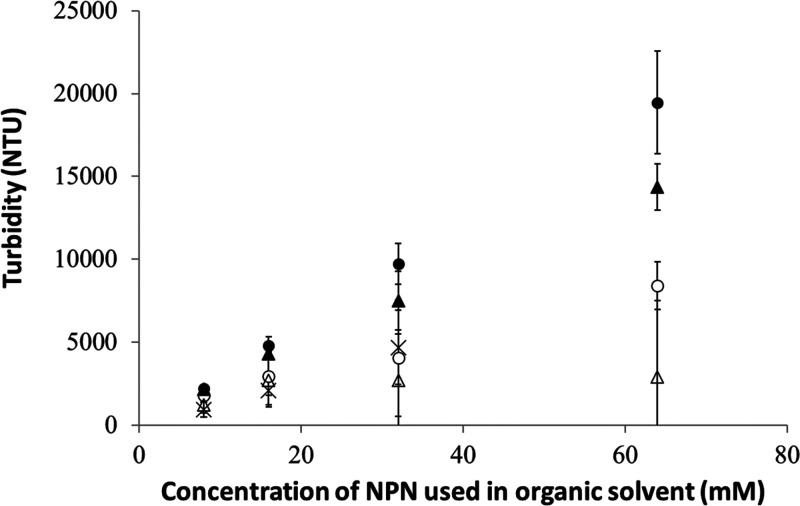
Turbidity quantification of NPN dispersions using known turbidity standards. Turbidity measurements are shown for initially prepared samples of NPN in various solvents and samples after 24 h. Values are shown as the average of true experimental triplicates for each concentration, with error bars representing ±1 standard deviation. The intensity of the light scattered is given in nephelometric turbidity units (NTU). DMSO at *t* = 0 (solid circles), DMSO at 24 h (empty circles), acetone at *t* = 0 (×), ethanol at *t* = 0 (solid triangles), and ethanol at *t* = 24 h (empty triangles).

To quantify the observations made on the cloudiness of these solutions (from Figure 1), turbidity measurements were performed versus a known turbidity standard (Figure 4). The light-scattering intensity of the starting dispersions upon dilution with water was linear. This allowed for the dilution of the dispersions to turbidities within a calibration curve using known turbidity standards to determine the NTU values for the undiluted samples. Previous studies using divinylbenzene/ethanol + water dispersions also showed that dilution of dispersions with water resulted in a proportional decrease in the number density of droplets.^2^

Consistent with expectation, turbidity measurements showed that the more concentrated samples scattered more light versus the lower concentration samples. This data also indicate that the turbidity is related linearly to the concentration of the NPN used and not the volume of solution added to create the dispersion. For example, the 64 mM sample at time zero is twice as turbid as the 32 mM sample, even though the same volumes of solutions were added (100 μL solution to 900 μL of water for a 10-fold dilution) to create the dispersions.

Turbidity measurements of these samples indicate that they are less turbid over time. After 24 h, the dispersions settled and required resuspension by vortex mixing before turbidity measurements could be done. The dispersions stayed uniformly suspended for longer than the duration of the measurements, and no immediate settling was seen. Since NPN is a water-insoluble solid, when a crystalline precipitate is formed, it can be seen either by eye or via microscopy. Samples after 24 h did not indicate the formation of any crystalline precipitate (data not shown) and instead were consistent in appearance with the original dispersion. This means that the decrease in turbidity was not due to a precipitation or bulk phase separation event. For each solvent, the decrease over time in turbidity is likely due to a decrease in the number of scatterers that are present. This conclusion is supported by particle size analysis also done herein. Interestingly, the samples that were prepared with NPN/acetone had a lower starting turbidity than DMSO samples of the same concentration of NPN. This can be seen visually as well when comparing the freshly prepared vials of NPN/DMSO to NPN/acetone; the NPN/acetone samples appear “less cloudy”. This again is likely a property of the scatterers present in the sample and was not indicative of a precipitation event.

The aforementioned turbidity measurements were done using concentrations of NPN similar to those used in virological studies. From this information, however, it is not clear what the minimum concentration is for the onset of dispersion formation. To elucidate the onset concentration, a series of dilutions was performed where various amounts of each solute–solvent (e.g., NPN-DMSO) were added to water for final varying concentrations of solute. This was performed for samples that were stable over the course of measurement as shown in Table 3. Graphical data that were used to determine the onset of dispersion formation can be found in Supporting Information. It can be seen from this data that the concentration for onset of dispersion formation is relatively similar for INAP and NPN in DMSO and acetone, both of which are polar aprotic solvents. The onset concentrations are lower for the ethanol dispersions for both INAP and NPN, suggesting that the intermolecular forces associated with the supersaturated polar-protic droplets are more stabilizing for the droplets that are formed. Additional samples were attempted using DMN, which created dispersions that were too unstable to measure with other techniques. It was seen that much higher concentrations in DMSO were needed to create an initial dispersion when compared to INAP/solvent and NPN/solvent dispersions. DMN dispersions phase-mixed within 5 min when added to acetone and ethanol, preventing measurements from being taken.

**Table 3:** Concentrations for the Onset of Dispersion as Measured by the Onset of Turbidity^a^

	Concentration for Onset of Dispersion (mg/mL)
INAP/DMSO	6.31 ± 2.28
INAP/acetone	6.87 ± 1.22
INAP/ethanol	4.99 ± 1.79
DMN/DMSO	30.80 ± 2.87
DMN/acetone	unstable
DMN/ethanol	unstable
NPN/DMSO	4.44 ± 1.11
NPN/acetone	6.16 ± 1.11
NPN/ethanol	3.74 ± 0.46

a Concentrations represent the amount of solute in the total aqueous dispersion volume. Measurements were taken in true triplicate. Graphical data used to determine these values can be found in the Supporting Information.

### Particle Size Determination

**Figure 5:**
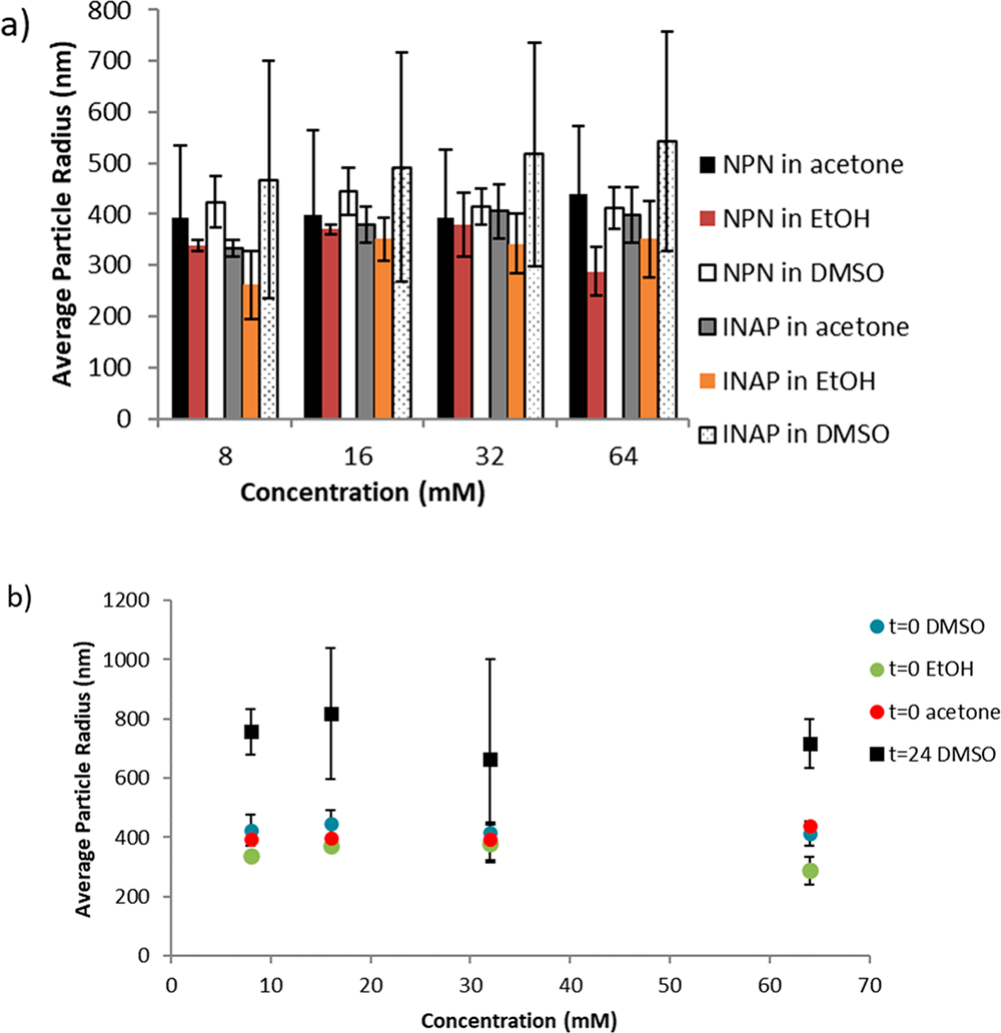
Particle sizes of colloids. Sizing measurements are shown for (a) initially prepared samples (*t* = 0) for both NPN and INAP in various solvents and for the (b) NPN/DMSO samples after storing for 24 h (*t* = 24) with comparison to starting values (*t* = 0) in various solvents. Values are shown as the average of true experimental triplicates for each concentration, with error bars representing ±1 standard deviation.

To obtain the full picture, particle sizing was performed using dynamic light-scattering (DLS) (Figure 5). The turbidity measurements shown in Figure 4 indicate a linear relationship between the turbidity and starting NPN concentration, where a higher starting concentration of NPN results in a greater turbidity. The data in Figure 5 indicate that the starting particle size at time zero is similar for all concentrations tested, regardless of whether DMSO or acetone was used and regardless of INAP or NPN. In other words, the turbidity in these dispersions is determined by the initial concentration of solute, whereas the initial particle size is not driven by the initial concentration. The particle size created with each concentration is constant, but the number of particles (and therefore scatterers) created is greater when larger concentrations are used, which results in the increase in turbidity that is seen with increasing concentrations (Figure 4). The driving force for the absolute size of these starting particles in this system is not clear, but it is presumably related to the rate of mixing (rapid vortex mixing) and the starting volume of solvent that is used, with the latter having an effect on the excess oil-to-solvent ratio.^2^ Further studies into the driving force for consistent initial particle size are ongoing.

Additionally, there is a marked difference in turbidity measurements between the NPN/DMSO and NPN/acetone samples at *t* = 0 (Figure 4). The intensity associated with light-scattering at 90° (used to measure turbidity) is dependent on the number of scatterers and the size of the scatterers. This suggests that either (1) there is a different number of initial scatterers or (2) the scatterers (particles containing NPN) are of a different size. Since the data in Figure 5 indicate the same particle size for all dispersions studied, they suggest that the number of scatterers for these solutions (NPN/DMSO and NPN/acetone) at comparable concentrations is different. One possible explanation for this is that the starting concentrations within the individual droplets that are formed are different for the NPN/acetone versus the NPN/DMSO samples. For example, if the initial solution used was 8 mM NPN/DMSO or 8 mM NPN/acetone, the droplets that are formed are likely not droplets of 8 mM solution but instead are droplets of kinetically stable supersaturated solution that is formed upon addition to water. This logically could vary between acetone and DMSO samples due to differences in the nature of these solvents’ polarity. Other studies show that the use of different solvents (for the systems studied) has varying effects on the size of the particles formed. Vitale et al. show that divinylbenzene (DVB)/ethanol and DVB/DMSO in water dispersions have the same particle sizes for the same DVB concentrations; however, some deviation from these particle sizes exists for DVB/acetonitrile in water dispersions, which they attribute to acetonitrile remaining in the dispersed phase.^2^ No comment is made, however, on the turbidity of these solutions as a function of solvent and therefore on the number of particles formed for the same concentrations. In fact, other studies only focus on the use of one solvent and variation of the concentration of solute/solvent in the dispersed phase.^3,5^ This suggests that keeping the solute concentration constant and varying the solvent are avenues for further study of these types of dispersions.

The growth in size of the particles in the NPN/DMSO samples over time (Figure 5) suggests a mechanism where smaller particles are combining to form larger particles, such as the case in Ostwald ripening,^3,6^ although under microscopic analysis, aggregation or coalescence of these particles is not readily observed as it happens. Microscopic analysis after 24 h does show an increase in the size of the droplets, and difficulties in measuring DLS data for the 24 h time-points for the NPN in acetone and INAP samples appear to be due to the presence of larger scatterers that irreproducibly affect the correlation function when DLS data is collected and push the limits of particle size determination for the instrument. The end result is the inability to determine the average particle radius for the NPN/acetone, INAP/acetone, and INAP/DMSO samples at *t* = 24 h. The NPN/DMSO samples at 24 h, as well as all the *t* = 0 samples, yielded reproducible data, with regularization fits that indicate monomodal distributions.

Overall, these colloids can be made reproducibly in water using NPN or INAP/acetone or NPN or INAP/DMSO, with DMSO having the advantage of added stability and colloidal persistence for longer time periods. Also, these colloids are not formed when NPN is added as a solid or INAP is added as a liquid to comparable mixtures of acetone/water and DMSO/water, indicating that favorable polar aprotic solvent–NPN interactions need to occur first to favor particle formation and to screen the formation of water–solvent interactions. This observation is consistent with the formation of “ouzo-like” dispersions.

## Conclusions

Metastable “ouzo-like” dispersions form when hydrophobic molecules are dissolved in a water-miscible solvent, which is then added to water. We have discovered that certain naphthalene-based compounds, *N*-phenyl-1-naphthylamine (NPN) and 1-iodonaphthalene (INAP)- readily form these dispersions without the need for added emulsifying or stabilizing agents. Dispersions of amorphous particles can be reproducibly created using dimethyl sulfoxide (DMSO), ethanol, or acetone, when NPN/DMSO, NPN/ethanol, NPN/acetone, INAP/DMSO, INAP/ethanol, or INAP/acetone mixtures are added to water. The dispersions made using DMSO solutions added to water were found to be stable for longer periods of time than the acetone or ethanol solutions of comparable concentration. The size of the particles is reproducible and comparable between DMSO, ethanol, and acetone, with an average radius of ∼400 nm when initially prepared. Turbidity measurements, when combined with particle-sizing DLS data, suggested that over time (days), Ostwald ripening occurred, followed eventually by heterogeneous nucleation resulting in precipitated NPN or phase separation of INAP. This unexpected property of these systems has not been explored previously and has implications in the delivery of hydrophobic molecules to biological membranes in experimental studies. For example, DMSO solutions of naphthyl compounds, similar to the one in this study,^13,16,17^ have been used for in vitro delivery of hydrophobic molecules to biological membranes within an aqueous environment, allowing for the hydrophobic molecule “payload” to be delivered, resulting in the molecule residing within the hydrophobic region of the bilayer membrane. Other LLPS systems^4^ have shown that the formation of the supersaturated droplets enhances the delivery of the solute to the membrane, suggesting that the ouzo-like system herein may also influence delivery of naphthalenic compounds to membranes. Our group is currently exploring the effect of the formation of these dispersions on the delivery of naphthyl-derived compounds to biological membranes.

## Supporting Information Available

Scattering data used to determine onset of dispersion (Table 3) values (PDF)

## Abbreviations

DLS: dynamic light scattering
DMSO: dimethyl sulfoxide
INAP: 1-iodonaphthalene
NPN: *N*-phenyl-1-naphthylamine
DMN: dimethoxynaphthalene
NTU: nephelometric turbidity units
