# Supplementary material for: Metastable Amorphous Dispersions of Hydrophobic Naphthalene Compounds Can Be Formed in Water without Stabilizing Agents via the “Ouzo Effect”
Source: J Phys Chem B. 2023 Sep 12;127(37):8032–9. doi: 10.1021/acs.jpcb.3c03885 (PMC10518816; doi:10.1021/acs.jpcb.3c03885)
Supplement: Supplementary file 1 — jp3c03885_si_001.pdf [file jp3c03885_si_001.pdf]

Supporting Information

Metastable Amorphous Dispersions of Hydrophobic Naphthalene Compounds Can be Formed in Water Without Stabilizing Agents Via the “Ouzo Effect”

Julie M. Belanger - *King’s College, Department of Chemistry and Physics, 133 N. River St., Wilkes-Barre, PA, 18711*

Joseph A. Cirilo, Jr. - *King’s College, Department of Chemistry and Physics, 133 N. River St., Wilkes-Barre, PA, 18711*

The following graphs contain the data used to determine the values for the onset of colloidal dispersion formation as given in Table 3 in the main article. The error bars represent the standard deviation between true experimental triplicates (ie new colloid samples were prepared and tested for each replicate).

a)

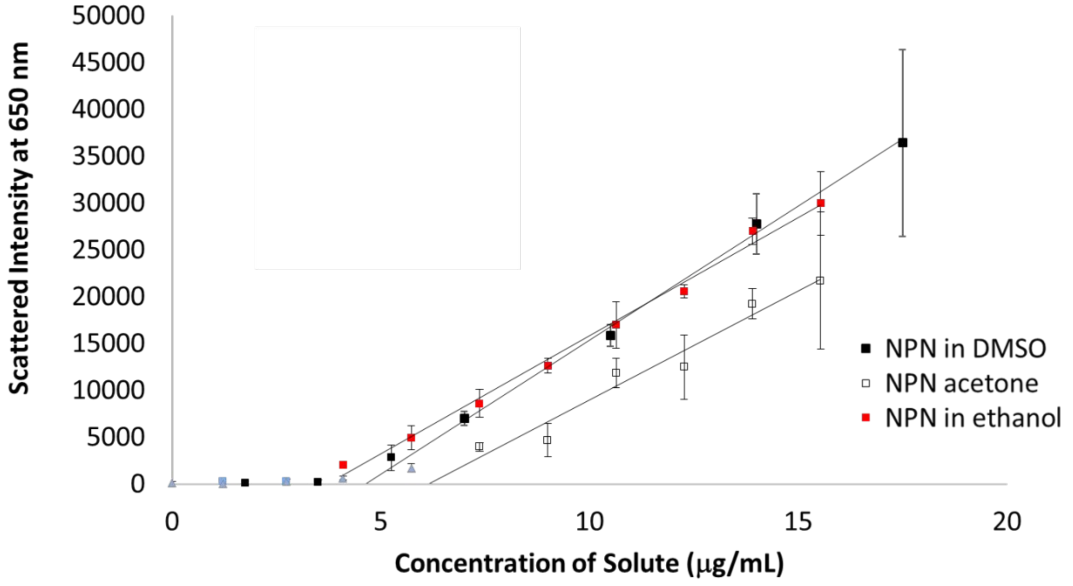

b)

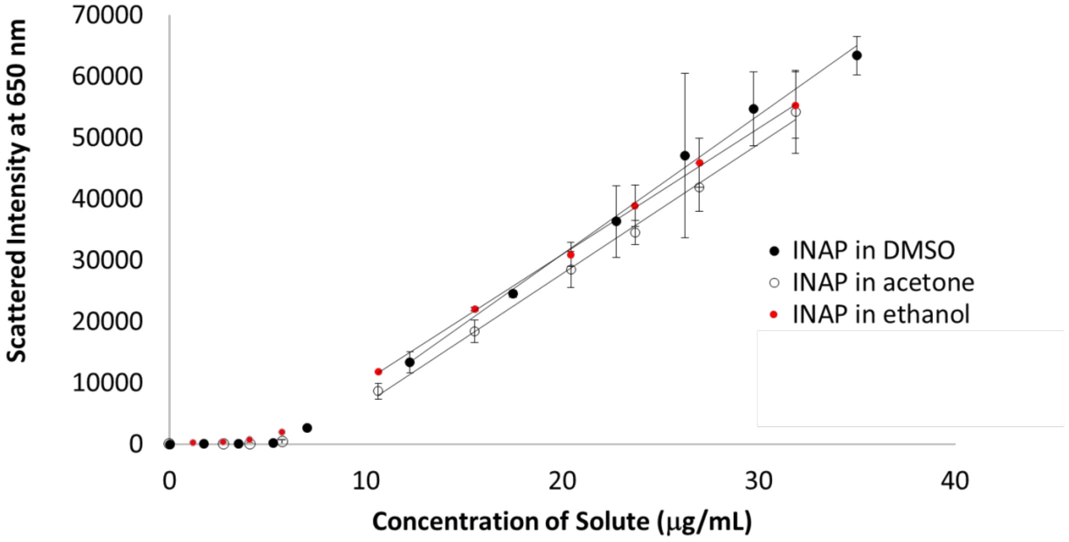
Scattered Intensity at 650 nm
Concentration of Solute (μg/mL)
INAP in DMSO
INAP in acetone
INAP in ethanol
0
10000
20000
30000
40000
50000
60000
70000
0
10
20
30
40

c)

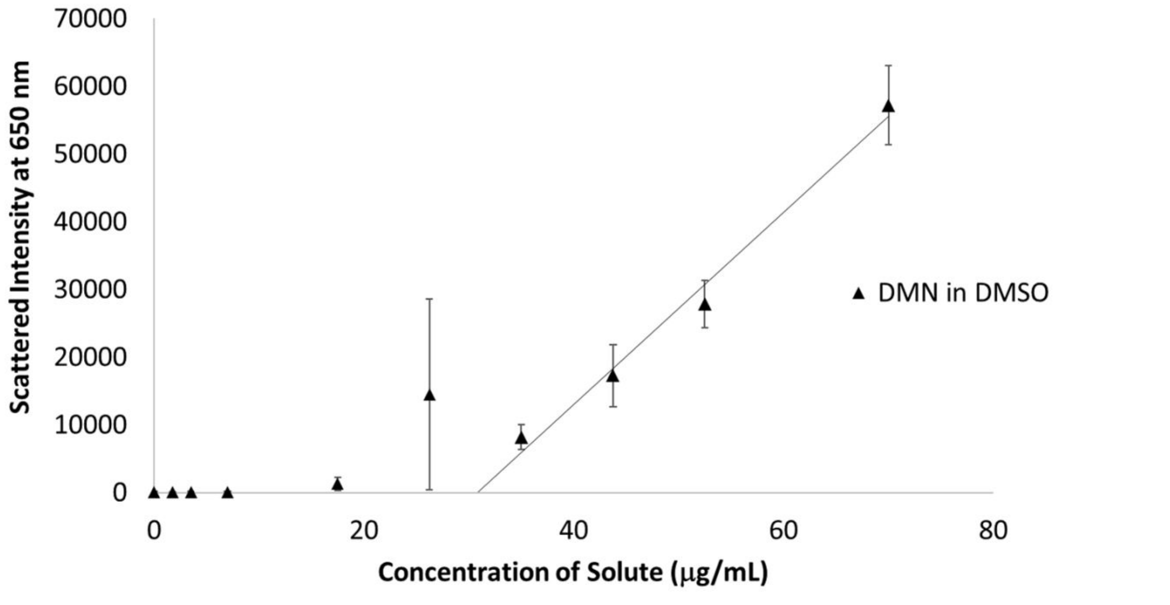
Scattered Intensity at 650 nm
Concentration of Solute (μg/mL)
DMN in DMSO
0
10000
20000
30000
40000
50000
60000
70000
0
20
40
60
80
